# Drowning at Sea

**Published:** 1885-12

**Authors:** 


### DROWNING AT SEA.

A person who will throw himself on his back in the water, with his hands held clasped in each other at his back, and with his head thrown back so that the nose and mouth may protrude from the water, may float for hours and cannot sink in that position.

A common feather pillow tied around under the arms is said to be worth half a dozen India rubber life preservers, while a common mattrass placed on a blanket, a trunk on the mattrass, then both trunk and mattrass tied up in the blanket and all thrown into the water together, will float with the tide for many hours.

DURING the last year bees in Ohio gathered 1,731,085 pounds of honey, estimated to be worth $276,975, while the fowls produced 32,602,321 dozen of eggs, valued at $4,890,348. The value of the egg was nearly equal to that of the wool produced in the State.

